# Practices of algorithm education based on discovery learning using a program visualization system

**DOI:** 10.1186/s41039-016-0041-5

**Published:** 2016-08-17

**Authors:** Koichi Yamashita, Ryota Fujioka, Satoru Kogure, Yasuhiro Noguchi, Tatsuhiro Konishi, Yukihiro Itoh

**Affiliations:** 1 Faculty of Business Administration, Tokoha University, 1230 Miyakoda, Kita-ku, Hamamatsu, Shizuoka 431-2102 Japan; 2 Graduate School of Informatics, Shizuoka University, 3-5-1 Johoku, Naka-ku, Hamamatsu, Shizuoka 432-8011 Japan; 3 Faculty of Informatics, Shizuoka University, 3-5-1 Johoku, Naka-ku, Hamamatsu, Shizuoka 432-8011 Japan; 4 Shizuoka University, 3-5-1 Johoku, Naka-ku, Hamamatsu, Shizuoka 432-8011 Japan

**Keywords:** Algorithm education, Learning support system, Domain world model, Classroom practice, Discovery learning

## Abstract

In this paper, we describe three practical exercises relating to algorithm education. The exercises are based on a learning support system that offers visualization of program behavior. Systems with the ability to visualize program behavior are effective to promote the understanding of algorithm behavior. The introduction of these systems into an algorithm course is expected to allow learners to cultivate a more thorough understanding. However, almost all existing systems cannot incorporate the teacher’s intent of instruction that may be necessary to accommodate learners with different abilities by using a different instructional approach. Based on these considerations, we conducted classroom practice sessions as part of an algorithm course by incorporating the visualization system we developed in our previous work. Our system visualizes the target domain world according to the visualization policy defined by the teacher. Our aim with the practical classes is to enable learners to understand the properties of algorithms, such as the number of comparisons and data exchanges. The contents of the course are structured such that the properties of an algorithm can be understood by discovery learning in the practical work. In this paper, we provide an overview of our educational practices and learners’ responses and show that the framework we use in our practices can be established in algorithm classes. Furthermore, we summarize the requirements for the inclusion of discovery learning in the algorithm classes as the knowledge obtained from our practices.

## Background

As computer education is recognized to be one of the fundamental aspects of science education, the range of educational opportunities for introducing students to algorithms has been expanded. A growing number of universities have implemented an algorithm course for non-computer science majors, because algorithm education is expected to foster logical thinking and develop general problem formulation and solving skills.

Generally, an algorithm course is held in the form of classroom lectures, and this is accompanied by a corresponding programming course held in the form of exercises to confirm and establish an improved understanding of the learning material covered by the lecture. In this way, learning by instruction in the lecture and establishing an understanding by exercising construct a learning cycle. The cycle enables learners to understand algorithms as the essence of problem solving and program code as the formal representation of algorithm externalization. However, according to our classroom experience, a number of learners are unable to develop the learning style required for this cycle and reach an impasse. We consider this to be the consequence of them proceeding to the programming exercise without sufficient understanding of the algorithm.

To date, several learning support systems have been developed to support novice learners to understand algorithms (Pears et al., 2007). These systems visualize the way objects of program code and the algorithm are processed (i.e., the target domain world) and reproduce the behavior of the code and the algorithm. Introducing these systems into classes is expected to allow learners to cultivate an improved understanding of algorithms (Robins et al., 2003).

However, the expansion of the range of educational opportunities has increasingly required teachers to teach learners with various levels of background knowledge. Depending on learners’ prior knowledge, a teacher would have to adjust the content or intent of instructions, such as the point where the learners should focus on the algorithm or program, or the abstraction or generalization degree of instruction. Almost all of the existing systems disallow these variations of the teacher’s intent and visualize the world of the target domain with a fixed visualization policy. Moreover, although existing systems tend to focus on reproducing the entire flow of algorithm behavior, knowledge related to the algorithm is also important in algorithm education. For example, properties of an algorithm such as the number of comparisons or exchanges in sorting tasks are an important learning target.

In this paper, we describe the classroom practice of our algorithm course, based on these considerations. We introduced the learning support system developed in our previous work (Kogure et al., 2014) into the class. Our system visualizes the target domain world according to the visualization policy defined by the teacher. In addition, the contents of our practice not only include using our system to learn about the entire flow representing the behavior of an algorithm but also discovery learning about the properties of the algorithm. Learners’ reactions suggest that it would be possible to successfully establish the framework of our practice in algorithm classes.

## Preceding work

### Basic idea

Systems supporting novice learners to learn an algorithm or program visualize its behavior in the target domain world. The learners understand the structure of the world and the principles to control the behavior in the world by using visualization. These systems include TRALKA2 (Malmi et al., 2004), Jeliot3 (Ben-Ari et al., 2011), ViLLE (Rajala et al., 2008), iList (Fossati et al., 2008), and LEPA (Yamashita et al., 2016). Learners understand the structure of the world by observing logical data structures visualized automatically by the system based on the provided algorithm or program code. Moreover, learners understand the algorithm or program code as the principles of behavior in the world by observing the changes in the status of the domain world as visualized in a step-by-step manner by the system.

**Fig. 1:**
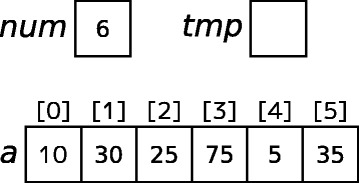
Example of the status of the target world

In many existing systems, the policies for visualizing the target domain world are pre-defined by the system developer. This may be an obstacle for a teacher who plans to introduce the system into actual classes. Teachers often explain various concepts by visualizations and change their policy of visualization depending on the learners. For example, a teacher may draw an array object using a horizontal layout when the instruction target is sorting of the array (as in Fig. 1), whereas the teacher may draw it by using a vertical layout for a stack. Changes such as these in the visualization policy are derived by adjusting the contents of the instruction to learners’ background knowledge. For example, once learners sufficiently understand a stack, representing an object using either a horizontal or vertical layout will be acceptable to them. Similarly, when teaching non-novice learners, the teacher would not need to draw the temporary variable in a task in which the values of two variables are exchanged.

Typically, visualized data structures are displayed to learners by showing them slides and/or movies produced with presentation and video editing software. Nevertheless, these materials cannot be used for certain learning activities, such as when learners are required to observe program behavior where input data are changed individually, because the input data are fixed. Other methods that would allow learners to change the input data would involve providing target program that includes graphic drawing functions. However, this would also not be realistic because it might burden teachers with troublesome coding, such as creating, updating, and deleting drawing objects with name resolution that involves namespaces, scopes, and so on.

**Fig. 2:**
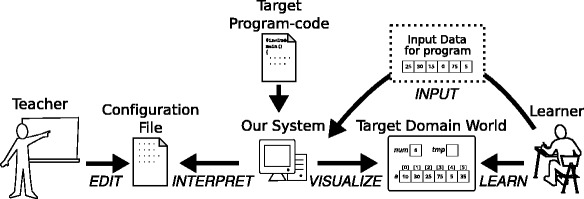
Relationship among teacher, learner, and our system

We resolved this problem by developing a learning support system that enables teachers to define the policy for drawing the status of the target domain world according to their own intent (Kogure et al., 2014). Our system reproduces the behavior of the program based on the definition. Teachers can define the visualization policy in a configuration file separate from the target program code, after which our system scans it, interprets the drawing policy, and visualizes the target domain world accordingly. This enables learners to observe the program behavior in the target world visualized in accordance with the teacher’s intent. The relationship among the teacher, learner, and our system is shown in Fig. 2.

### Defining a visualization policy for the target domain world

A configuration file that defines the visualization policy consists of a set of drawing rules, each of which contains the following information:

- Condition to actuate the drawing operation
- Operation to edit the target drawing object
- Identifier of the target drawing object
- Type of target drawing object
- Attributes for the drawing operation

We selected the available object and attribute types by examining actual teaching materials for algorithm and programming education in the faculties to which the authors are affiliated. Table 1 provides a list of the available types, operations, and attributes. Circle, square, and rectangle objects are mainly used to express directly the value of the variable in the target program. The table object mainly expresses an array. Connector and line objects express a relationship between the two objects, and label and balloon objects are used to describe program behavior or the role of an object in natural language.

**Table 1:** Types of objects, drawing operations, and attributes for configuration

Objects	Operations	Attributes
Circle	Create	Corresponding variable^a^
Square	Delete	Main object ID^b^
Rectangle	Update	Position
Table		Width
Connector		Height
Line		Color
Label		Line color
Balloon		String color

^a^Only for circle, square, rectangle, and table objects

^b^Only for connector, line, and balloon objects

Teachers can specify a condition for actuating the drawing operation by referencing the statement number (statement ID) or variables in the target program, such as “when a certain statement is executed,” or “when the value of a certain variable satisfies the condition.” A condition can be expressed with six types of comparison operators, ==, !=, >=, <=, <, and >, and three types of operands, i.e., the immediate number, a variable in the target program, and the statement ID.

**Fig. 3:**
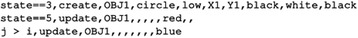
Examples of drawing rule descriptions in the configuration file

Some examples of drawing rules that include such information are provided in Fig. 3 in CSV format. The description in the first line is a rule that determines that, when the statement with ID “3” in the target program is executed, our system draws a circle object and assigns the object ID “OBJ1” to it. The corresponding variable is described as “*low*,” and hence the value of the variable *low* is drawn inside OBJ1. OBJ1 is drawn at position (“X1,” “Y1”) with a line, background, and inner character colors of “black,” “white,” and “black,” respectively, according to the description in the rule. The rule in the second line means that when the statement with ID “5” is executed, the line color of the already visualized object “OBJ1” is updated to “red.” The last line is the rule that when variable “*j*” has a value that is greater than “*i*” in the target program, the inner character color of “OBJ1” is updated to “blue.”

### Overview of learning environment produced by our system

**Fig. 4:**
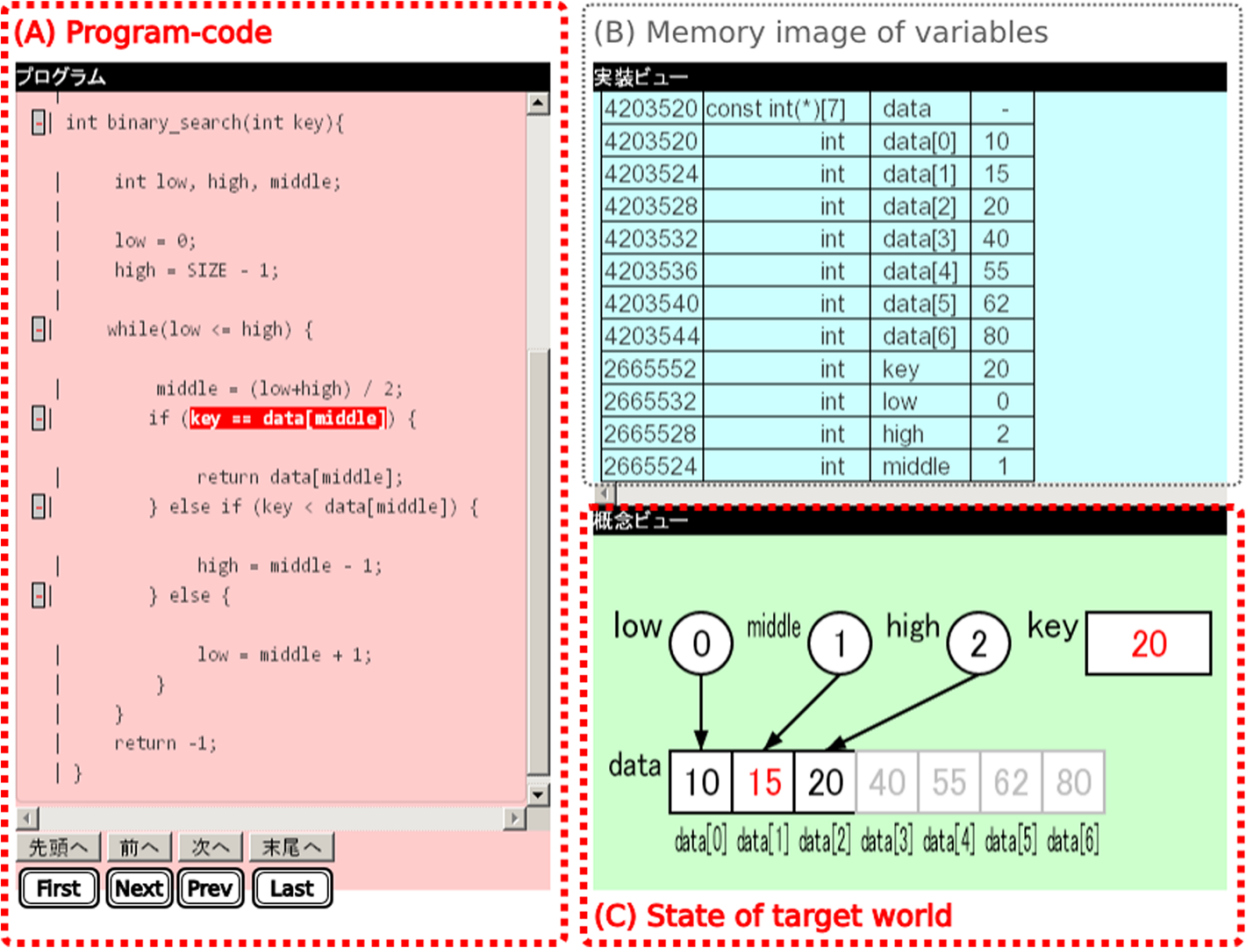
Overview of learning environment produced by our system

Figure 4 shows a learning environment generated by our system. The environment consists of three fields: the data structures processed by the program in (A) are visualized in the two fields in (B) and (C). Our system reproduces a series of memory images of variables in (B) for each step of the execution of the program and a series of statuses of the target domain world in (C) that visualizes logical data structures. The definitions in the configuration file represent the policy to visualize the world in (C). When a learner clicks the “Next” or “Prev” button, the highlight in (A) moves to the next or previous statement in the program code, whereupon the memory image in (B) is updated according to the values of the variables after executing the highlighted statement, and the corresponding status of the target domain world is visualized in (C). Our system simulates the statement execution step by step so that the learner can understand the program behavior by observing the changes in the target domain world in (C). Moreover, if there are some connector objects or descriptions with label and balloon objects reflecting instructions in a class, they will assist the learner to understand the program behavior. Field (B) represents the status of the memory image, which is implemented for the learning target that needs to reference the main memory, such as a pointer.

The learning environment in Fig. 4 is an instance for observing the behavior of a binary search program. We needed to describe 31 drawing rules to define the visualization in (C) for this environment.

### Preliminary experiment

We conducted a small preliminary experiment in order to evaluate the difficulty of preparing teaching material by the rule definitions in our system. The subjects in the experiment were one Master’s student and two undergraduate students with their capacity to perform the role as teaching assistant. We measured the time required for the subjects to prepare teaching materials by using the rule definitions in the following manner: first, we provided each subject with sufficient tutorials to enable them to produce teaching materials by the rule definitions in our system. Next, we presented the subjects with the program code for selection sort and requested them to describe the rule set required to visualize the program behavior in the target domain world. Our request included conveying that each subject has to implement the visualization policy pre-established by us. After completing the rule definition, we requested the subjects to create slide materials using PowerPoint with the same content and behavior as the materials produced by the rule definitions. Table 2 provides the times required for each subject to complete each procedure.

**Table 2:** Times required to complete each procedure

	Subject A	Subject B	Subject C
Tutorial	56 min	43 min	52 min
Rule definitions	32 min	30 min	33 min
Slide creations	23 min	33 min	29 min

Although the number of subjects was small, the result of this experiment suggests that teaching materials for our system could be prepared in practical time with some experience in rule definitions. The times required to complete the rule definitions are approximately the same as those for slide creations. However, this rule-based teaching material would be more useful because our system can reproduce the program behavior without rule modifications, even if the target data processed by the program were to change.

### Related works

The ANIMAL (Rössling and Freisleben, 2002) is mentioned as a system with similar functions to our system. In the context of learning with ANIMAL, users of the system visualizing the behavior of programs are regarded as taking on one of the four different roles defined in (Price et al., 1998):

1. User/viewer, agent observing the target domain world visualized by the system.
2. Visualizer/animator, agent defining the visualization of the target domain world with the system.
3. Software developer, agent developing the visualization system.
4. Programmer, agent designing the algorithm or program code that is the target of visualization.

The ANIMAL is a system that aims to achieve overall improvement in terms of learning support by providing sophisticated support for each of the four above-mentioned roles, by straightening the differences among them. In the context of learning with our system, learners take on the user/viewer role and the teacher takes on the visualizer/animator and programmer roles. The software developer role is taken on by us.

The visualizer/animator in ANIMAL needs to use a script language named AnimalScript to define the visualization of the target domain world. Although the description capability increases significantly by using the script language, the cost associated with learning the language is a matter that cannot be ignored. Moreover, the amount of script required to define the visualization in ANIMAL is generally larger than the amount of visualization rules used in our system. For example, the sample script for a bubble sort algorithm bundled in ANIMAL consists of 170 lines of code, whereas our configuration file for bubble sort consists of 56 lines of rule. Rössling and Ackermann (2007) attempted to reduce the defining costs by bundling some ready-to-use sample scripts together with a GUI front-end for arranging the scripts. Likewise, our system is being extended to enable the visualizer/animator to use a front-end GUI to define the visualization rules.

## Classroom practice sessions

In existing systems including ANIMAL, an action in the domain world is implemented by the target program code or algorithm rather than by the users; that is, users’ GUI operations are used mainly to observe changes in the domain world. In contrast, our system provides a means of data input into the target program by learners themselves. Data input into the target program implements an action to the domain world without the need to edit the program code. We consider the use of this function to introduce discovery learning into algorithm education. Below in this section, we describe three classroom practical exercises with our system based on this consideration.

The first practical exercise involves fundamental sorting algorithms, incorporated into actual classes, and conducted in order to confirm that an algorithm class including discovery learning could be established as a form of algorithm education. The second includes search algorithms and was conducted separately from the actual classes, confirming that our framework of practice could be applied to classes other than those covering sorting algorithms. The third was another about sorting algorithms, was incorporated into the actual classes covering the same topic, and was conducted to investigate the effects of our framework. In each practical exercise, we planned learning without referencing the memory image in (B). Participants referred to field (A) as the formal expression of the algorithm, from which we omitted the syntax details. Participants also focused on the observations in field (C) to understand the behavior of the algorithms. Table 3 provides a brief summary of our classroom practice sessions.

**Table 3:** Summary of our classroom practice sessions

	Class #1	Class #2	Class #3
No. of participants	24	4	19
Target algorithm	Sorting	Search	Sorting
Actual course	Incorporated	Not incorporated	Incorporated
Learning time	90 + 90 min	120 min	90 + 90 min
Test	1 pre + 1 post	1 post only	3 pre + 3 post
Years and month	July 2014	Feb 2015	July 2015

### Class #1: sorting algorithms

In the faculty to which one of the authors is affiliated, the lecture course “algorithm” is held for third year students with a business administration major. We incorporated our above-mentioned system into two of the classes in the course. The practical classes covered three fundamental sorting algorithms, namely selection sort, insertion sort, and bubble sort. The goal of these classes was to achieve an understanding of the behavior of the three sorting algorithms and to achieve an understanding of the differences among them based on the number of comparisons and swaps. We planned to accomplish the former goal by using our system to enable learners to observe the changes in the target domain world and the latter goal by using tasks based on discovery learning that find the maximum and minimum number of comparisons and swaps. There were 24 participants in this practical class, all of whom were business administration majors, 21 years old, with less than 1 year of programming experience.

In the first practical class, the teacher who regularly taught the course lectured on the three sorting algorithms for 75 min. At the end of the class, we conducted a 15-min pre-test to evaluate the learners’ understanding of the three sorting algorithms before using our system. The pre-test required learners to determine the values of variables by tracing the algorithms. In the second class, which was conducted a week later, the teacher explained how to operate the system containing our learning environment and how to observe the target domain world in the first ten minutes. After that, we allowed learners to use our environment to explore and learn about each of the sorting algorithms. The learning process involved the following steps.

1. The learners observe the changes in the target domain world for the program code as a whole and confirm the behavior of the algorithm.
2. The teacher makes the learners aware of the executions of comparison and swap operations, and suggests that the number of these operations could be varied according to the initial order of the array.
3. The learners input the initial order of the array into our environment, and confirm the variation in the number of comparisons and swaps.
4. The teacher requests the learners to find the initial order of the array that maximizes and minimizes the respective number of comparisons and swaps.
5. The teacher inquires of the learners their discovery of the initial array order and explains the property of the target algorithm based on the learners’ replies.

Although the order in which the three algorithms can be presented to learners is arbitrary, we introduced the environments in the order of bubble sort, insertion sort, and selection sort in these practical classes. We omitted steps 2 and 3 above when presenting insertion and selection sort. The task in step 4 is based on discovery learning, hence we required the learners to follow this step for all three algorithms. Step 4 consists of the following sub-steps that construct the discovery learning cycle:

- Learners generate a hypothesis about the array order that satisfies the requirements.
- Learners input the array order based on their hypothesis in 4a.
- Learners verify the hypothesis by observing the behavior of the algorithm using the array order in 4b.
- Learners return to step 4a and regenerate a more plausible hypothesis as necessary based on the verification results in 4c.

We incorporate the input process in the target program code for realizing steps 3 and 4 to enable learners to input the initial order of the array when tracing the program. After the entire 60-min learning period devoted to the three algorithms, we conducted a 15-min post-test to evaluate learners’ understanding of the three sorting algorithms after using our system. The post-test consisted of tasks involving tracing the same algorithms as in the pre-test except for the target array.

The teacher created three configuration files to enable learners to learn by following the above-mentioned steps before the practical classes. The configuration files consisted of 56, 65, and 62 drawing rules for bubble sort, insertion sort, and selection sort, respectively. Many of the drawing rules are defined in order to enable the target domain world to be visualized in a way similar to which existing systems visualize these worlds. Meanwhile, some of the rules are defined to visualize the teacher’s intents such as ensuring that the instruction contents correspond to a learner’s background knowledge and providing guidance during the discovery process. Specifically, the intents of instruction included in our environment by the teacher were the following:

- To suggest the role of the variables to learners by drawing an arrowed connector from the variables indexing the array to the corresponding array elements.
- To clarify the difference between sorted and unsorted elements by coloring sorted elements only.
- To clarify the difference of a role by drawing circle objects for the index variables, with a rectangle object for the temporary variable in swaps.
- To suggest the viewpoints in discovery learning by using label objects to notify the execution of comparison and swap.

**Fig. 5:**
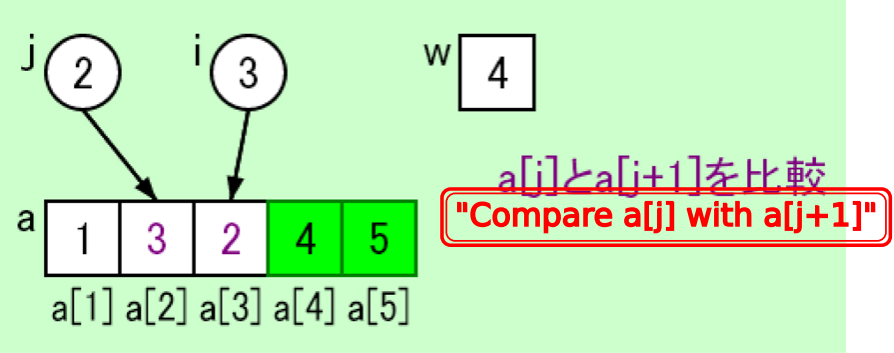
Example of the status of the target domain world for a sorting algorithm

All of these intents of instruction were suggestive rather than codified, because we intended to encourage learners to focus on the number of comparisons and swaps by themselves in step 4. Figure 5 provides an example of the status of the target domain world visualized by our system.

Both the pre- and post-tests elicited values for all the variables on the specified watch points in a series of processes for each algorithm. The teacher scored the tests on an all or none basis, i.e., one point for correct answers and zero for wrong answers for every value at every watch point. The pre-test and post-test contained 64 and 56 questions, respectively. Table 4 presents the scores of the two tests. We placed the scoring rates in parentheses to allow for a comparison between both tests and for comparison with the class to be mentioned later. We determined whether the difference in the scoring rate between the pre- and post-test is statistically significant by performing a two-sided independent t-test with a significance level of 5 %. The calculation showed *t*(23) = 3.98, *p* < 0.01; hence, we found the difference to be significant.

**Table 4:** Pre- and post-test scores of each participant in class #1

ID	Pre-test	Post-test	Difference
1	7 (0.11)	24 (0.43)	(0.32)
2	23 (0.36)	39 (0.70)	(0.34)
3	27 (0.42)	33 (0.59)	(0.17)
4	0 (0.00)	0 (0.00)	(0.00)
5	24 (0.38)	23 (0.41)	(0.04)
6	41 (0.64)	45 (0.80)	(0.16)
7	64 (1.00)	56 (1.00)	(0.00)
8	0 (0.00)	0 (0.00)	(0.00)
9	10 (0.16)	20 (0.36)	(0.20)
10	0 (0.00)	0 (0.00)	(0.00)
11	19 (0.30)	24 (0.43)	(0.13)
12	0 (0.00)	0 (0.00)	(0.00)
13	0 (0.00)	3 (0.05)	(0.05)
14	0 (0.00)	0 (0.00)	(0.00)
15	0 (0.00)	0 (0.00)	(0.00)
16	32 (0.50)	40 (0.71)	(0.21)
17	25 (0.39)	56 (1.00)	(0.61)
18	22 (0.34)	28 (0.50)	(0.16)
19	50 (0.78)	55 (0.98)	(0.20)
20	0 (0.00)	0 (0.00)	(0.00)
21	14 (0.22)	42 (0.75)	(0.53)
22	0 (0.00)	0 (0.00)	(0.00)
23	20 (0.31)	20 (0.36)	(0.05)
24	31 (0.41)	31 (0.55)	(0.15)
Ave.	16.3 (0.26)	22.5 (0.40)	(0.14)

Note that there are no participants with a lower post-test rate than a pre-test rate. Participants with no difference in their rates are those who either obtained a full score or a zero score. Although the scoring rate of all participants increased by 0.14 on average, those who had pre-test scores greater than zero increased the rate by 0.22 on average.

### Class #2: search algorithms

We conducted another practical session for fundamental search algorithms separate from the actual classes during which instruction was provided. Search algorithms had not been introduced during the time of instruction because of the shortage of time. Three search algorithms were introduced during this class: sequential search, binary search, and hash search. The goal of the class was to allow learners to develop an understanding of the behavior of and the differences among the three search algorithms based on the search key and the search target. We planned to achieve the goal by observing the changes in the target domain world using our system and by the tasks based on discovery learning. The tasks assessed whether learners were able to find the maximum and minimum number of comparisons and the synonym records. Four learners participated in this practical session, all of whom were business administration majors, 21 years old, and had less than 1 year of programming experience. All of them had participated in the practical session described in the previous subsection and none had any learning experience in search algorithms.

The practice class was of 120-min duration. The teacher first explained how to operate our learning environment and how to observe the target domain world and then conducted the class in the following steps:

1. The teacher provides the learners with the definition of the search problem. The teacher then explains that the class focuses on searching from an array rather than from a file and encourages the learners to think about a search algorithm spontaneously.
2. The teacher introduces the basic idea of a sequential search algorithm as one of the simplest solutions and allows the learners to observe its behavior using our environment.
3. The teacher requests the learners to input some search keys and observe the change in the number of comparisons and then requires the learners find the search key that maximizes and minimizes the number of comparisons.
4. The teacher inquires of the learners their discovery of the search key and consequently explains the property of a sequential search algorithm, naïve but inefficient, based on their replies.
5. The teacher mentions that there are more search algorithms, introduces the basic idea of a binary search algorithm, and allows the learners to observe its behavior using our environment.
6. The teacher requests the learners to input some search keys, observe the change in behavior, and find the search key that maximizes and minimizes the number of comparisons.
7. The teacher inquires of the learners their discovery of the search key and consequently explains the property of a binary search algorithm, fast but requires the search space to be sorted, based on their replies.
8. The teacher introduces the basic idea of a hash search as yet another algorithm, and allows the learners to observe the behavior of a hash search from a dataset with no synonyms using our environment.
9. The teacher allows the learners to observe the behavior of generating a hash table using a certain dataset and observe the occurrence of collisions. Here, the collision resolution of the algorithm used in the practical session is based on open addressing.
10. The teacher inquires of the learners why collisions occur and how the searching process changes. Then, the teacher requests the learners to find the dataset and search keys that trigger collisions.
11. The teacher encourages learners to report what they have discovered and what they have realized, and explains the properties related to a hash search such as hash function requirements and the size of hash tables, based on learners’ replies.
12. The teacher allows the learners to use our environment to observe the behavior of a hash search from a dataset with synonyms.

The configuration files are created by the teacher before the class, as in class #1. The configuration files consisted of 28 drawing rules for sequential search, 33 rules for binary search, 30 rules for hash search from a dataset with no synonyms, 41 rules for hash table generation, and 36 rules for hash search from a dataset with synonyms. The intent of instruction included in our environment by the teacher for reasons other than the visualization of elemental objects in the target domain world was the following:

- To suggest the role of variables to learners by drawing an arrowed connector from the variables indexing the array to the corresponding array elements.
- To clarify the difference in the roles by drawing circle objects for the index variables and a rectangle object for the search key.
- To clarify the difference between the search range and other array elements by coloring only out-of-range elements when using the sequential and binary search algorithms.
- To suggest the viewpoints in discovery learning by coloring two objects that are the target of the comparison process.

**Fig. 6:**
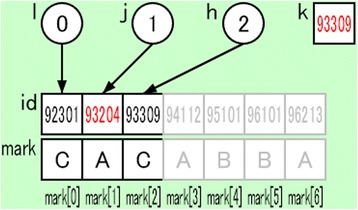
Example of the status of the target domain world for a binary search algorithm

We intended to support learners’ discovery experience by providing suggestive visualizations similar to those in the practice session described in the previous subsection. The tasks based on discovery learning are included in steps 3, 6, and 10. Figure 6 provides an example of the status of the target domain world when the binary search algorithm is visualized by our system. The search key is stored in the variable *k*. The search range is presented by using gray color for the array elements that are out of the search range. A comparison between two objects is presented by using red color for the target of the comparison process, 93204 and 93309. These visualizations suggest the objects on which the learners should focus and support the learners’ discovery learning.

After the practice session, we instructed the learners to write a briefing paper on search algorithms by briefly explaining their behavior and properties as a 15-min post-test. The teacher examined their responses from the viewpoint of whether the learners adequately described the three algorithms and their properties based on what they learned in this practice session. Consequently, we found that, although the learners were able to describe all three algorithms and roughly explain the behavior and properties of each, they tended to use informal terms in their descriptions. We consider the learners to have grasped the behavior and properties of the algorithms but have not acquired the terminology, because this practice did not include an instruction session.

### Class #3: sorting algorithms

The last practice class among the three was conducted in July 2015. The contents and goal of the class were the same as for class #1; that is, to understand the behavior of three sorting algorithms and to understand the differences among them based on the number of comparisons and swaps. We incorporated class #3 into the actual course “algorithm” as two consecutive sessions. We also included the same intent of instruction as those in class #1 in the configuration files. Learners followed steps 1 to 5 of class #1 when learning about each of the sorting algorithms with our environment. This time, 19 learners participated in the practice sessions, all of whom were business administration majors, 22 years old, with less than 1 year of programming experience. Four of them had participated in class #1 but had received zero for both of the pre- and post-tests.

Class #3 differed from class #1 in two respects. First, we used questionnaires to obtain leaner feedback at the end of the second practice session. We discuss these results in Section 4.Second, whereas the pre- and post-tests in class #1 required the values of variables to be obtained by tracing the three algorithms, those administered in class #3 consisted of the following three questions:

1. Asks the values of variables obtained by tracing the single provided algorithm in order to check the overall understanding of algorithm behavior.
2. Asks the number of comparisons and swaps to determine whether the learners could find the correct number for the provided algorithm and initial array.
3. Asks which algorithm is the most efficient to sort the provided initial array and why, to establish whether the learners could explain the differences among the algorithms appropriately.

The algorithms provided in Q1 and Q2 were different, as were the initial array provided in Q2 and Q3. These also differed between the pre-test and post-test.

The scoring was done by the teacher as in class #1. Q1 required the values of all variables on ten specified watch points in a series of processing the algorithm. The teacher scored one point for correct values at every watch point and zero for answers including the wrong value for Q1. Every answered number of comparisons and swaps for Q2 scored one point for each correct answer and zero if the answer was wrong. The teacher scored each answer in response to Q3 using a five-point scale, examined from the viewpoints as to whether the algorithm in the answer is the most efficient one, the description contains comparisons among the algorithms, the comparative perspective is appropriate, and the description contains a sufficient degree of details.

Table 5 presents the scores of the pre- and post-tests that were administered in class #3. We again provide the scoring rates in parentheses. We performed a two-sided independent *t* test with a significance level of 5 % for every question to determine whether the difference of each scoring rate between the pre- and post-tests is statistically significant. The calculation showed *t*_Q1_(18) = 3.71, *t*_Q2_(18) = 3.52, *t*_Q3_(18) = 2.39, and *p*_Q1_ < 0.01, *p*_Q2_ < 0.01, and *p*_Q3_ < 0.03; hence, we found all differences to be significant. Supposing that the averages of the scoring rates represent the entire scores of the pre- and post-test, the resultant scores largely have a similar tendency to those of class #1. We also performed a two-sided independent *t* test with a significance level of 5 % on all the averages of the scoring rates. The calculation showed *t*(18) = 4.22, *p* < 0.01, and hence we found the difference of the entire set of scoring rates to be significant. There was only one participant with a post-test rate lower than their pre-test rate. The average improvement in the scoring rate was 0.29, which suggests the effectiveness of the framework in our practical work.

**Table 5:** Pre- and post-test scores of each participant in Class #3

ID	Pre-test				Post-test				Difference			
	Q1	Q2	Q3	Ave.	Q1	Q2	Q3	Ave.	Q1	Q2	Q3	Ave.
1	1 (0.10)	0 (0.00)	1 (0.00)	(0.03)	0 (0.00)	2 (1.00)	3 (0.50)	(0.50)	(-0.10)	(1.00)	(0.50)	(0.47)
2	1 (0.10)	1 (0.50)	1 (0.00)	(0.20)	0 (0.00)	2 (1.00)	1 (0.00)	(0.33)	(-0.10)	(0.50)	(0.00)	(0.13)
3	0 (0.00)	2 (1.00)	1 (0.00)	(0.33)	0 (0.00)	1 (0.50)	1 (0.00)	(0.17)	(0.00)	(-0.50)	(0.00)	(-0.17)
4	0 (0.00)	0 (0.00)	1 (0.00)	(0.00)	0 (0.00)	1 (0.50)	1 (0.00)	(0.17)	(0.00)	(0.50)	(0.00)	(0.17)
5	0 (0.00)	0 (0.00)	1 (0.00)	(0.00)	0 (0.00)	0 (0.00)	1 (0.00)	(0.00)	(0.00)	(0.00)	(0.00)	(0.00)
6	5 (0.50)	0 (0.00)	1 (0.00)	(0.17)	10 (1.00)	2 (1.00)	5 (1.00)	(1.00)	(0.50)	(1.00)	(1.00)	(0.83)
7	0 (0.00)	1 (0.50)	1 (0.00)	(0.17)	5 (0.50)	1 (0.50)	3 (0.50)	(0.50)	(0.50)	(0.00)	(0.50)	(0.33)
8	1 (0.10)	2 (1.00)	1 (0.00)	(0.37)	6 (0.60)	1 (0.50)	3 (0.50)	(0.53)	(0.50)	(-0.50)	(0.50)	(0.17)
9	0 (0.00)	0 (0.00)	1 (0.00)	(0.00)	0 (0.00)	0 (0.00)	1 (0.00)	(0.00)	(0.00)	(0.00)	(0.00)	(0.00)
10	0 (0.00)	0 (0.00)	1 (0.00)	(0.00)	0 (0.00)	1 (0.50)	1 (0.00)	(0.17)	(0.00)	(0.50)	(0.00)	(0.17)
11	4 (0.40)	1 (0.40)	3 (0.50)	(0.47)	10 (1.00)	2 (1.00)	1 (0.00)	(0.67)	(0.60)	(0.50)	(-0.50)	(0.20)
12	4 (0.40)	0 (0.00)	3 (0.50)	(0.30)	10 (1.00)	2 (1.00)	5 (1.00)	(1.00)	(0.60)	(1.00)	(0.50)	(0.70)
13	3 (0.30)	0 (0.00)	1 (0.00)	(0.10)	5 (0.50)	2 (1.00)	3 (0.50)	(0.67)	(0.20)	(1.00)	(0.50)	(0.57)
14	0 (0.00)	0 (0.00)	1 (0.00)	(0.00)	0 (0.00)	0 (0.00)	1 (0.00)	(0.00)	(0.00)	(0.00)	(0.00)	(0.00)
15	0 (0.00)	0 (0.00)	1 (0.00)	(0.00)	5 (0.50)	1 (0.50)	1 (0.00)	(0.33)	(050)	(0.50)	(0.00)	(0.33)
16	0 (0.00)	0 (0.00)	1 (0.00)	(0.00)	10 (1.00)	2 (1.00)	5 (1.00)	(1.00)	(1.00)	(1.00)	(1.00)	(1.00)
	(0.00)	(0.00)	(0.00)	(0.00)	(1.00)	(1.00)	(1.00)	(1.00)	(1.00)	(1.00)	(1.00)	(1.00)
17	0 (0.00)	0 (0.00)	1 (0.00)	(0.00)	0 (0.00)	1 (0.50)	1 (0.00)	(0.17)	(0.00)	(0.50)	(0.00)	(0.17)
18	0 (0.00)	0 (0.00)	1 (0.00)	(0.00)	5 (0.50)	1 (0.50)	1 (0.00)	(0.33)	(0.50)	(0.50)	(0.00)	(0.33)
19	0 (0.00)	0 (0.00)	1 (0.00)	(0.00)	5 (0.50)	0 (0.00)	1 (0.00)	(0.17)	(0.50)	(0.00)	(0.00)	(0.17)
Ave.	(0.10)	(0.18)	(0.05)	(0.11)	(0.37)	(0.58)	(0.26)	(0.41)	(0.27)	(0.39)	(0.21)	(0.29)

## Discussion

### Discovery learning of the properties of an algorithm

Our intension was to enable learners to discover the properties of an algorithm rather than the algorithm itself because of the goal of the actual algorithm course. The course is expected to use case learning to promote general problem solving skills, rather than develop the fundamental skills required for program design. Hence, the teacher has placed great emphasis on instilling knowledge about existing algorithms. The evaluation results of Class #1 and #3 based on the pre- and post-test suggest that the learners cultivated an improved understanding of sorting algorithms through the practice sessions. The briefing papers written in Class #2 suggest that the learners understood the behavior of a search algorithm to some extent, despite being unsuccessful in mastering the terminology. We consider these results to confirm that the framework implemented in our practice sessions can be established in algorithm classes.

During coding exercises or when learning with a learning support system, learners develop the learning activity individually; hence, they would focus on irregular and inconsistent points for discovery. Kirschner et al. (2006) has pointed out that unguided learning is ineffective based on examples from science and medical education, and insisted on the necessity of guidance in discovery learning. Thus, we included visual objects in the visualized domain world, which serves as a guide for discovery. Not only does our system provide objects consisting of the target domain world but also suggestive viewpoints to reduce the irregularity and inconsistency. The visualizations defined in our practice exercises are not intended to explain the role of each object or the behavior of the algorithm, rather to suggest points on which the learners should focus, that is, to provide guidance for discovery learning. In our practical classes, participants replied actively by sharing their discovery with the teacher. Therefore, we consider our visualization to work effectively with the desired effect in terms of discovery learning.

### Factors influencing score improvements

Class #1 and #3 had an interval of 1 week between the pre- and post-test because of schedule restrictions. This was because we incorporated each of the practice exercises into a series of actual classes. These intervals are not short and consequently allowed the learners to learn out-of-class with textbooks and course notes. In this section, we discuss the questionnaire administered in class #3 and the survey results to evaluate the extent to which out-of-class learning influences the post-test score.

The questionnaire covered the following six aspects:

1. Enquires about the amount of out-of-class preparation and reviews using a five-point scale.
2. Enquires about the level of confidence in programming skills using a five-point scale.
3. Enquires about the level of interest in computer science using a five-point scale.
4. Enquires about the usability of our learning environment using a five-point scale.
5. Enquires about the learning target that cultivated an improved understanding.
6. Enquires about the learner’s impression or suggestions to improve our environment.

We included items E5 and E6 for the purpose of applying qualitative analysis, but unfortunately, most of the participants left these items unanswered. Table 6 provides the answers of each participant for E1 to E4.

**Table 6:** All answers on questionnaire in class #3

ID	E1	E2	E3	E4
1	3	2	4	4
2	1	3	4	4
3	2	2	4	2
4	1	1	2	3
5	1	1	1	3
6	3	2	4	5
7	1	1	1	4
8	2	3	4	3
9	1	1	2	3
10	2	2	3	4
11	2	2	2	3
12	1	1	3	5
13	1	1	2	5
14	2	2	3	4
15	2	2	4	3
16	2	3	4	5
17	3	2	3	4
18	2	2	4	4
19	4	2	3	4

**Fig. 7:**
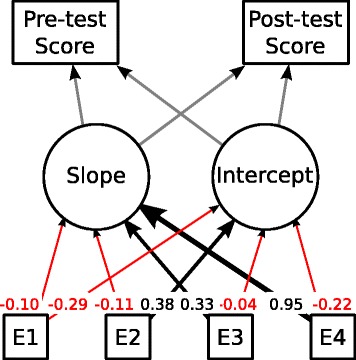
Path diagram representing our model in questionnaire analysis

We estimated the relationships between the scoring rate improvements in the pre- and post-tests and answers on the questionnaire by applying the following growth model: Both test rates were modeled as linear functions to obtain the distribution of slopes and intercepts. The slopes and intercepts serve as dependent variables, and questionnaire items serve as independent variables. We examined how each questionnaire item (i.e., the independent variables) affects both test scores (i.e., dependent variables) by applying regression analysis. Figure 7 provides an excerpt of our analysis. The numerical values accompanying the arrows in the diagram represent the partial regression coefficients which are standardized to a scale in the range [−1, 1]. The rectangle and circle nodes represent independent and dependent variables, respectively. We excerpt the numerical values concerning the following discussions for simplicity. Our model revealed a good fit to the data (GFI = 1.00, AGFI = 1.00).

According to Fig. 7, learning out-of-class (asked in E1) has a weak negative relation to both the slopes and intercepts. This can be interpreted that the slopes refer to the scoring rate improvements between pre- and post-tests and the intercepts refer to the pre-test rates, roughly. Based on the interpretation, both of the score improvements and pre-test scores had been influenced very little by out-of-class learning. That is, although there was an interval of one week between Class #1 and #3 because of schedule restriction, we can ignore the influence of the interval on the scores of the pre- and post-test. This evaluation result does not have sufficient reliability for generality because we could not procure a sufficient number of participants for the practice sessions. However, at least in Class #3 the score improvements would be influenced little by out-of-class learning.

Questionnaire items intended to probe effects other than out-of-class learning also had the expected outcome. The confidence in programming skills (E2) were found to have weak relations to intercepts. This suggests that the pre-test scores would be tangible confidence of programming skills. The interests in computer science (E3) have weak relations to slopes, suggesting that participants with an interest in computer science would be eager to attempt the classroom practice with the program visualization system. Evaluations of the usability of our system (E4) have an extremely strong relation to slopes. We think it is natural that participants who cultivated an improved understanding of algorithms graded the usability higher.

## Conclusion

In this paper, we described the educational practices based on the learning support system we developed in our previous work. The system, which offers visualization of program behavior, supports learners to understand algorithms, and hence introducing these systems is expected to allow learners to cultivate an improved understanding. However, existing systems tend to focus on reproducing the entire flow of the algorithm behavior, which means that it is necessary to structure the contents of classes such that the properties of an algorithm such as the number of comparisons or swaps are taught. Furthermore, almost all of existing systems disallows variation in the teacher’s intent of instruction to accommodate the learners. Based on these considerations, we introduced the system developed in our previous work. Our system visualizes the target domain world according to the visualization policy defined by the teacher. We included contents based on discovery learning about the properties of algorithms in our classroom practices. The teacher made learners externalize their discovery by inquiries and explained knowledge based on their externalized discovery.

We conducted three classroom practical sessions introducing our system. The evaluation results of the pre- and post-test in class #1 and #3 provided that the learners cultivated an improved understanding through our practices. The results of the post-test of class #2 showed that the learners gained an understanding of the general behavior of algorithms to a certain degree, though they gained little understanding of the terminology. These results suggest that the framework of our practices can be established in algorithm classes. Although we must consider that a statistically insufficient number of learners participating in the practice sessions may influence the accuracy of verification, we believe that continuous practice will suppress this reliability matter.

One of the limitations of our framework is that it is difficult to encourage adoption of the terminology such as the name of an algorithm. The transfer of knowledge in lecture classes would require the teacher to balance the lecture and time learners are allowed to practice with the system. We plan to find ideal material for use in classes by continuously conducting classroom practice sessions with our system. Further, the questionnaire administered in class #3 had inquiries concerning mainly the learner’s attributes; hence, our analysis based on the answers could not reveal factors that have (or do not have) a learning effect. We intend to continue improving the questionnaire inquiries in order to determine the direction for improving our class design and our system. Other future work is to conduct a more quantitative evaluation of the learning effects and efficiency of our classroom practices. We also plan to continue investigating how efficiently the framework in our practices is prepared and how effectively and efficiently the algorithm classes are conducted.

Our practices suggest that if the algorithm course is held as part of fundamental science education, the teacher could conduct the classes based on discovery learning by introducing an appropriate learning support system. We can summarize the requirements of classroom practice for algorithms including discovery learning such as our practice described in this paper as follows:

- Teacher needs to introduce the learning support system into the class for use by each of the learners individually.
- The system needs the functionality to allow the design of actions to the target domain world and user interface to apply actions other than coding.
- The system needs the functionality to construct the target domain world based on the teacher’s intent to suggest focusing points and support the learner’s discovery.

## References

[CR1] Ben-Ari M, Bednarik R, Ben-Bassat Levy R, Ebel G, Moreno A, Myller N, Sutinen E (2011). A decade of research and development on program animation: the Jeliot experience. Journal of Visual Languages & Computing.

[CR2] Fossati, D, Eugenio, BD, Brown, C, Ohlsson, S. (2008). Learning linked lists: experiments with the iList system. *Proceedings of the 9th International Conference on Intelligent Tutoring Systems*, 80-89. doi:10.1007/978-3-540-69132-7_13.

[CR3] Kirschner P, Sweller J, Clark RE (2006). Why unguided learning does not work: an analysis of the failure of discovery learning, problem-based learning, experiential learning and inquiry-based learning. Educational Psychologist.

[CR4] Kogure, S, Fujioka, R, Noguchi, Y, Yamashita, K, Konishi, T, Itoh, Y. (2014). Code reading environment according to visualizing both variable’s memory image and target world’s status. *Proceeding of the 22nd International Conference on Computers in Education*, 343-348.

[CR5] Malmi L, Karavirta V, Korhonen A, Nikander J, Seppälä O, Silvasti P (2004). Visual algorithm simulation exercise system with automatic assessment: TRAKLA2. Informatics in Education.

[CR6] Pears A, Seidman S, Malmi L, Mannila L, Adams E, Bennedsen J, Devlin M, Paterson J (2007). A survey of literature on the teaching of introductory programming. ACM SIGCSE Bulletin.

[CR7] Price B, Beacker R, Small I, Stasko J, Domingue J, Brown MH, Price BA (1998). An introduction to software visualization. Software Visualization.

[CR8] Rajala T, Laakso MJ, Kaila E, Salakoski T (2008). Effectiveness of program visualization: a case study with the ViLLE tool. Journal of Information Technology Education.

[CR9] Robins A, Rountree J, Rountree N (2003). Learning and teaching programming: a review and discussion. Computer Science Education.

[CR10] Rössling G, Ackermann T (2007). A framework for generating AV content on-the-fly. Electronic Notes in Theoretical Computer Science.

[CR11] Rössling G, Freisleben B (2002). ANIMAL: a system for supporting multiple roles in algorithm animation. Journal of Visual Language & Computing.

[CR12] Yamashita K, Nagao T, Kogure S, Noguchi Y, Konishi T, Itoh Y (2016). Code-reading support environment visualizing three fields and educational practice to understand nested loops. Research and Practice in Technology Enhanced Learning.

